# Antimelanogenic Effects of *Polygonum tinctorium* Flower Extract from Traditional Jeju Fermentation via Upregulation of Extracellular Signal-Regulated Kinase and Protein Kinase B Activation

**DOI:** 10.3390/ijms19102895

**Published:** 2018-09-24

**Authors:** You Chul Chung, Ji-Hye Ko, Hyun-Kyu Kang, Seoyeon Kim, Choon Il Kang, Jung No Lee, Sung-Min Park, Chang-Gu Hyun

**Affiliations:** 1 Department of Chemistry and Cosmetics, Jeju National University, Jeju 63243, Korea; jyc8385@hanmail.net (Y.C.C.); asdfg12309@daum.net (J.-H.K.); superxyz1993@naver.com (H.-K.K.); asksy613@naver.com (S.K.); 2 JejuIndi Inc., Seogwipo-si, Jeju 63635, Korea; indi0701@daum.net; 3 R&D Center, CoSeedBioPham Co., Chungbuk 28161, Korea; jnlee2000@hanmail.net (J.N.L.); smp@coseed.co.kr (S.-M.P.)

**Keywords:** *Polygonum tinctorium*, nuruk, ERK, antimelanogenesis, whitening, fermentation

## Abstract

This study was carried out to investigate the antimelanogenic effects of a *Polygonum tinctorium* flower extract obtained using red nuruk, a traditional Jeju barley-based fermentation starter. We also studied the mechanism of action of the *P. tinctorium* fermented flower extract (PTFFE) in mouse melanoma cells (B16F10). Cells were treated with various concentrations (62.5, 125 and 250 μg/mL) of PTFFE and the results showed that PTFFE significantly decreased the melanin content and tyrosinase activity without being cytotoxic. In addition, PTFFE strongly inhibited the expression of tyrosinase and tyrosinase-related protein 2 by decreasing the expression of the microphthalmia-associated transcription factor, as shown by a western blot assay. Furthermore, PTFFE inhibited melanogenesis via upregulation of the phosphorylation of extracellular signal-regulated kinase (ERK) and protein kinase B, also known as AKT. We also used inhibitors such as PD98059 (a specific ERK inhibitor) or LY294002 (an AKT inhibitor) to determine whether the signaling pathways are involved. High-performance liquid chromatography fingerprinting showed the presence of a quercetin glucoside (isoquercitrin) and quercetin in PTFFE. To test the potential for PTFFE application as a cosmetic material, we also performed a primary skin irritation test on human skin. In this assay, PTFFE did not induce any adverse reactions at the treatment dose. Based on these results, we suggest that PTFFE may be considered a potential antimelanogenesis candidate for topical applications.

## 1. Introduction

Melanin determines the skin color and its primary role is to prevent skin damage from harmful ultraviolet (UV) radiation. However, excessive production of melanin causes skin diseases such as freckles, melisma, lentigo and other hyperpigmentation syndromes [1,2]. Thus, studies on inhibition of melanogenesis have focused on medical and cosmetic treatments for skin depigmentation and whitening. Tyrosinase acts as a very important enzyme, catalyzing the oxidation of l-tyrosine as well as l-3,4-dihydroxyphenylalanine (l-DOPA) to DOPAquinone. DOPAquinone thus formed, undergoes intramolecular cyclization to form leucoDOPAchrome, which is further oxidized to DOPAchrome [3].

Recent studies have also indicated that the microphthalmia-associated transcription factor (MITF) and melanogenic enzymes such as tyrosinase-related proteins 1 and 2 (TRP-1 and TRP-2) contribute to melanogenesis in B16F10 cells. MITF binds to the M-box in the tyrosinase promoter and then regulates the expression of tyrosinase and other melanogenic enzymes such as TRP-1 and TRP-2. Accordingly, it has recently been reported that hyperpigmentation is prevented by inhibition of MITF [4,5,6].

Mitogen-activated protein kinases (MAPKs), a family of serine/threonine kinases, including p38, extracellular signal-regulated kinase (ERK) and c-Jun N-terminal kinase (JNK), are important factors in melanin synthesis. Recent studies have reported that MAPKs are involved in MITF regulation [7,8,9]. Suppression of p38 and JNK phosphorylation reduces the expression of MITF and melanogenic enzymes that lead to melanogenesis [10,11]. In addition, transcription of tyrosinase, which activates melanin synthesis, is decreased by the activation of ERK phosphorylation [12]. Furthermore, activation of the phosphatidylinositol 3-kinase (PI3K)/protein kinase B (also known as AKT) signaling pathway has been reported to increase the phosphorylation of MITF and decrease the tyrosinase expression, thereby downregulating the melanin synthesis [13].

Recently, researchers have developed tyrosinase inhibitors and biological reductants such as kojic acid, arbutin, hydroquinone and ascorbic acid to treat hyperpigmentation and pigmentation diseases [14,15]. However, the use of whitening agents that can inhibit tyrosinase is limited because of their cytotoxicity, low stability in the presence of oxygen and water and side effects such as skin irritation and dermatitis [16,17]. Therefore, development of safer and more effective agents for skin whitening is still continued in the field of cosmetics research and development.

Nuruk, a fermentation starter, has been used for brewing traditional wines in Korea [18,19]. Nuruk contains many types of microorganisms, such as fungi, yeasts and some bacteria. These microorganisms are responsible for saccharification and alcohol fermentation, respectively [20,21]. A recent study has reported that the process of fermentation has the potential to improve biological properties of the raw material by producing new beneficial compounds [22]. Red nuruk, a traditional Jeju barley-based fermentation starter, has been used for brewing a traditional wine (Makgeolli) or fermented drink (Shindari) in Jeju only; however, no research on the red nuruk fermentation has been reported yet. Therefore, we examined the effect of a red nuruk-fermented *Polygonum tinctorium* flower extract (PTFE) on melanogenesis in B16F10 cells.

*Persicaria tinctoria* (Ait.) H. Gross an annual herb, is a species of flowering plants in the buckwheat family, whose leaves and stems have been used as a medicine [23]. It is used in the treatment of freckles, pimples, epidemics, infantile convulsions and high febrile conditions in children [24]. A recent study has reported that PTFE could exert antioxidant, anti-wrinkle, whitening and UV-protective effects [25]. However, *P. tinctorium* fermented flower extract (PTFFE) has not been studied yet. Therefore, in this study, α-melanocyte-stimulating hormone (α-MSH)-stimulated B16F10 cells were treated with PTFFE to investigate the effects of PTFFE on the expression of melanogenic enzymes.

## 2. Results

### 2.1. Effects of PTFFE on the Viability of B16F10 Cells

**Figure 1:**
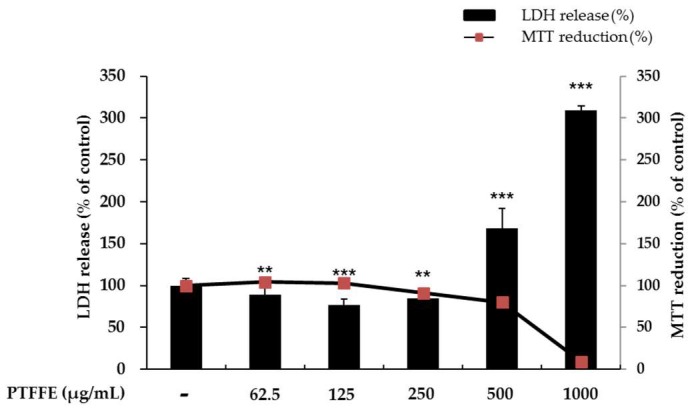
Effects of *P. tinctorium* fermented flower extract (PTFFE) on the viability of B16F10 melanoma cells. The cells were treated with PTFFE at the indicated concentrations for 48 h. Cell viability is expressed as a percentage relative to the value for the untreated cells. The data are presented as the mean ± standard deviation (SD) of at least three independent experiments. ** *p* < 0.01, *** *p* < 0.001.

Cell viability was evaluated using a 3-(4,5-dimethylthiazol-2-yl)-2,5-diphenyltetrazolium bromide (MTT) assay. To determine whether PTFFE shows cytotoxicity against B16F10 melanoma cells, cells were treated with various concentrations of PTFFE (62.5, 125, 250, 500 and 1000 μg/mL) for 48 h. As shown in Figure 1, there were no significant effects on the cell viability at PTFFE concentrations between 62.5 and 250 μg/mL, relative to the untreated control cells. The cell viability decreased by 21% and 90% in the groups treated with 500 and 1000 μg/mL PTFFE, respectively. Therefore, we used PTFFE concentrations of 62.5, 125 and 250 μg/mL for further experiments.

To evaluate if the selected concentrations of PTFFE could cause cellular damage and cytolysis, the lactate dehydrogenase (LDH) released to the extracellular medium was spectrophotometrically quantified. It was observed that PTFFE 500 and 1000 μg/mL induced LDH release (60% and 220% variation respectively to untreated cell), while other concentrations of PTFFE did not alter LDH release relatively to the untreated cell. Due to cellular toxicity induced by the high concentrations of PTFFE (500 and 1000 μg/mL), these concentrations were not considered for the present study.

### 2.2. Effects of PTFFE on Melanin Production

**Figure 2:**
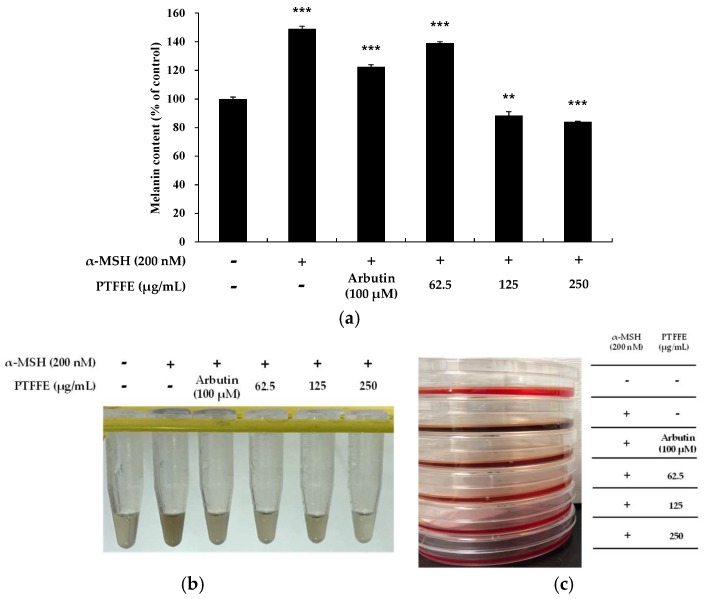
Effects of PTFFE on melanin production in B16F10 melanoma cells. Cells were treated with PTFFE at the indicated concentrations for 72 h. α-MSH (200 nM) and arbutin (100 μM) were used as a stimulator and a positive control, respectively. The melanin content is expressed as a percentage relative to the value in the untreated cells. The data are presented as the mean ± SD of at least three independent experiments (**a**). ** *p* < 0.01, *** *p* < 0.001. The dissolution of the cell pellet and the photograph of the culture medium were shown in (**b**,**c**), respectively.

To examine the effects of PTFFE on melanin synthesis, we determined the melanin content in cells treated with various concentrations of PTFFE for 72 h. α-MSH (200 nM) and arbutin (100 μM) were used as a positive and negative control, respectively. As shown in Figure 2, PTFFE treatment decreased the melanin content in a dose-dependent manner. In particular, PTFFE at 250 μg/mL inhibited the intracellular melanin content by 43% compared with that in the group treated with positive control, which was 16% lower than the melanin content in the untreated group.

### 2.3. Effects of PTFFE on Intracellular Tyrosinase Activity

**Figure 3:**
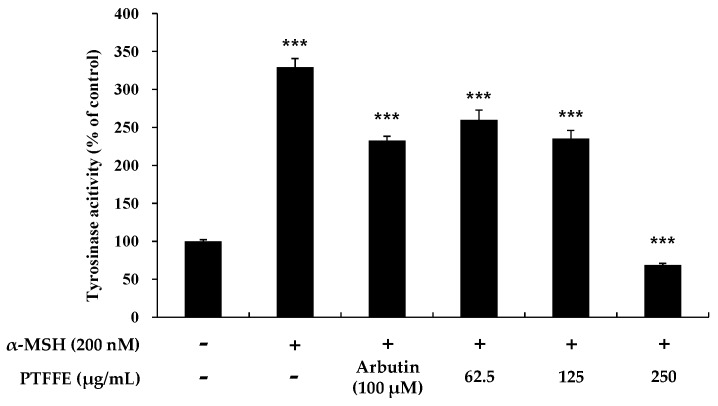
Effects of PTFFE on tyrosinase activity. B16F10 melanoma cells were treated with PTFFE at the indicated concentrations for 72 h. α-MSH (200 nM) and arbutin (100 μM) were used as a positive and negative control, respectively. The results are expressed as percentages of the untreated cells. The data are presented as the mean ± SD of at least three independent experiments. *** *p* < 0.001.

To clarify whether PTFFE affects melanogenesis via inhibition of tyrosinase activity, we examined the intracellular tyrosinase activity in PTFFE-treated B16F10 melanoma cells. As shown in Figure 3, tyrosinase activity was inhibited by PTFFE at all the concentrations tested compared with that in the positive control group. In particular, at 250 μg/mL, PTFFE decreased tyrosinase activity to 32% compared with untreated control, which was 290% lower than that in the 200 nM α-MSH-treated group.

### 2.4. Effects of PTFFE on the Expression of Melanogenic Enzymes and MITF in B16F10 Cells

**Figure 4:**
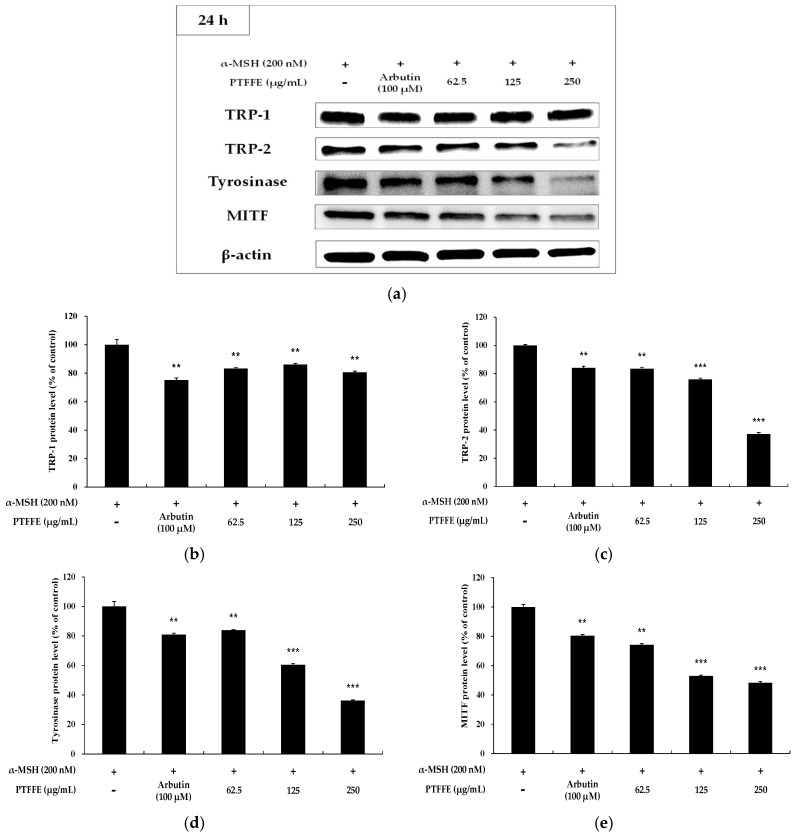
Effects of PTFFE on TRP-1, TRP-2, tyrosinase and MITF expression in B16F10 cells. Cells were treated with the indicated concentrations of PTFFE for 24 h. Protein levels were determined by western blotting. (**a**) Western blotting results and protein levels of (**b**) TRP-1, (**c**) TRP-2, (**d**) tyrosinase and (**e**) MITF. Results are expressed as percentages of the positive control. The data are presented as the mean ± SD of at least three independent experiments. ** *p* < 0.01, *** *p* < 0.001.

To determine the effects of PTFFE on the expression of MITF and melanogenic enzymes, western blots analysis was performed on B16F10 cells treated with various concentrations of PTFFE. As shown in Figure 4, the expression of TRP-2 and tyrosinase in B16F10 cells was significantly decreased by PTFFE treatment. Moreover, the MITF expression decreased in a concentration-dependent manner, which suggested that PTFFE inhibits melanogenic enzymes by decreasing the MITF expression.

### 2.5. Effects of PTFFE on the AKT Signaling Pathway

**Figure 5:**
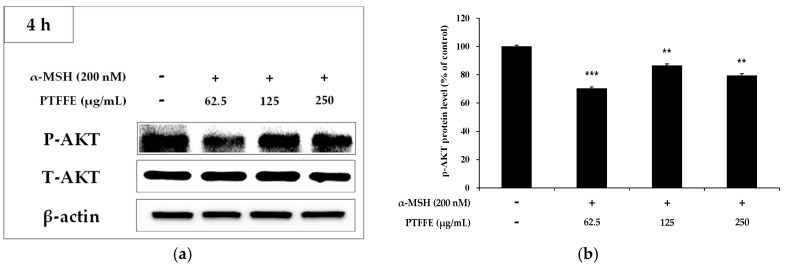
Effects of PTFFE on AKT phosphorylation. B16F10 cells were treated with the indicated concentrations of PTFFE for 4 h. (**a**) Protein expression levels were investigated by western blotting. (**b**) Results are expressed as percentages of the untreated cells. The data are presented as the mean ± SD of at least three independent experiments. ** *p* < 0.01, *** *p* < 0.001. P: phosphorylated, T: total.

**Figure 6:**
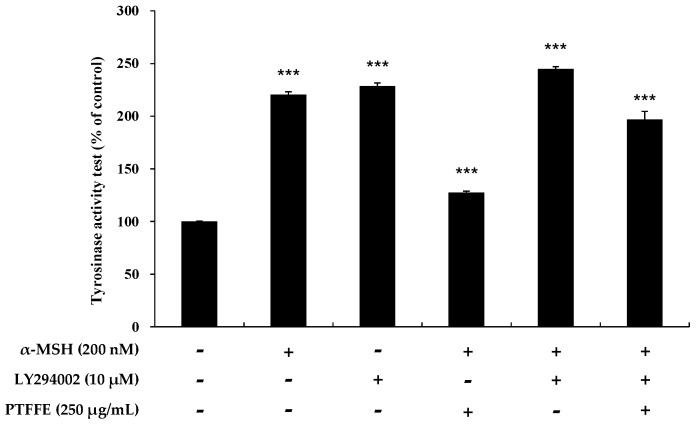
Effects of PTFFE and the AKT inhibitor LY294002 on tyrosinase activity in B16F10 cells. Cells were treated with α-MSH (200 nM) or LY294002 (10 μM) alone or in combination and with or without PTFFE at the single concentration of 250 μg/mL. Data are expressed as percentages of the untreated cells. The data are presented as the mean ± SD of at least three independent experiments. *** *p* < 0.001.

Activation of the AKT signaling pathway has been reported to be involved in the inhibition of melanin synthesis [13]. Therefore, upstream signaling pathways potentially involved in the hypopigmentary effects of PTFFE were investigated. As shown in Figure 5, treatment of B16F10 cells with PTFFE at 62.5 and 250 μM enhanced the AKT phosphorylation. Next, we examined the effect of LY294002 (an AKT inhibitor) on tyrosinase activity to investigate whether AKT activation is involved in melanogenesis. As shown in Figure 6, tyrosinase activity induced in B16F10 cells by α-MSH or LY294002 was reduced by PTFFE treatment. These results indicated that PTFFE inhibits tyrosinase activity in B16F10 cells through the AKT signaling pathway, thus effectively suppressing the melanin production.

### 2.6. Effects of PTFFE on MAPK Phosphorylation in B16F10 Cells

**Figure 7:**
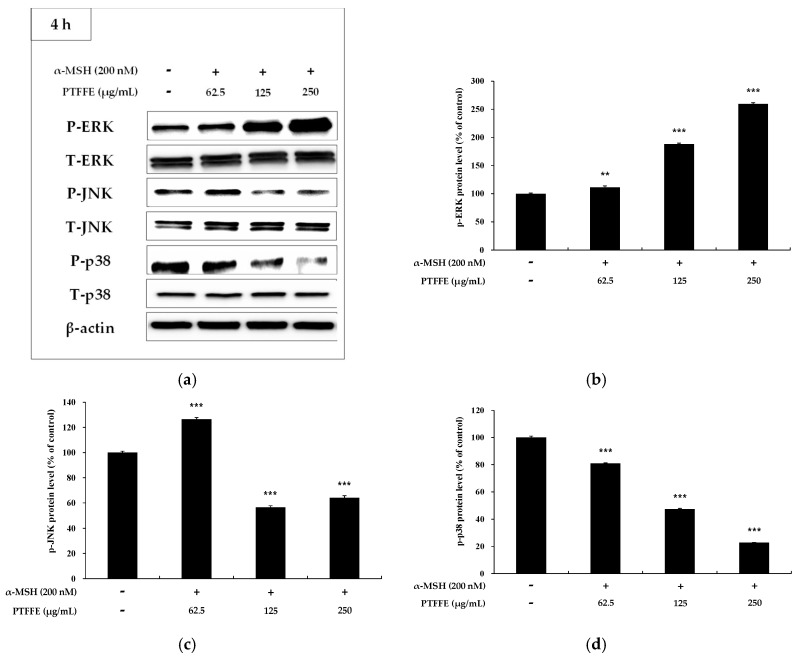
Effects of PTFFE on phosphorylation of ERK, p38 and JNK. B16F10 cells were treated with PTFFE at the indicated concentrations for 4 h. (**a**) Western blotting results and protein levels of (**b**) p-ERK, (**c**) p-JNK and (**d**) p-p38. Data are expressed as percentages of the untreated cells. The data are presented as the mean ± SD of at least three independent experiments. ** *p* < 0.01, *** *p* < 0.001. P: phosphorylated, T: total.

**Figure 8:**
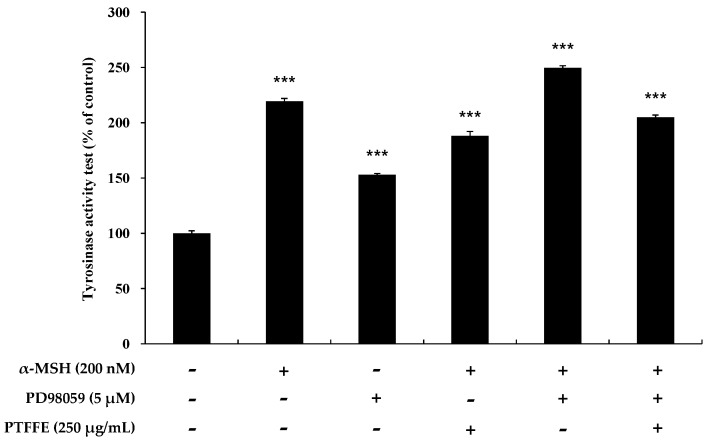
Effects of the ERK inhibitor PD98059 on tyrosinase activity in B16F10 cells. Cells were treated with α-MSH (200 nM) or PD98059 (5 μM) alone or in combination and with or without PTFFE at the single concentration of 250 μg/mL. Data are expressed as percentages of the untreated cells. The data are presented as the mean ± SD of at least three independent experiments. *** *p* < 0.001.

To determine whether PTFFE affects the MAPK phosphorylation, western blot analysis was performed on B16F10 cells treated with various concentrations of PTFFE. As shown in Figure 7, PTFFE dramatically increased phosphorylation of ERK and decreased that of JNK and p38. To determine whether an increase in ERK phosphorylation downregulates the tyrosinase production, treatment with a specific inhibitor of the ERK pathway, PD98059, was performed. As shown in Figure 8, the tyrosinase activity induced in B16F10 cells by α-MSH or PD98059 was inhibited by PTFFE treatment. These results indicate that PTFFE inhibits melanogenesis by increasing the ERK phosphorylation and inhibiting that of JNK and p38 in B16F10 cells.

### 2.7. Human Skin Primary Irritation Test of PTFFE

To evaluate whether PTFFE can potentially be used as a component in cosmetics, a human skin irritation test was carried out by using a patch with PTFFE on the human back. As shown in Table 1, PTFFE did not cause any particular irritation at 30 min and 24 h after the patch was removed. In particular, we did not observe severe side effects, such as erythema, burning, or pruritus. These results suggest that PTFFE is a safe raw material for the application as a cosmetic ingredient.

### 2.8. Isoquercitrin and Quercetin Contents in PTFE and PTFFE

**Figure 9:**
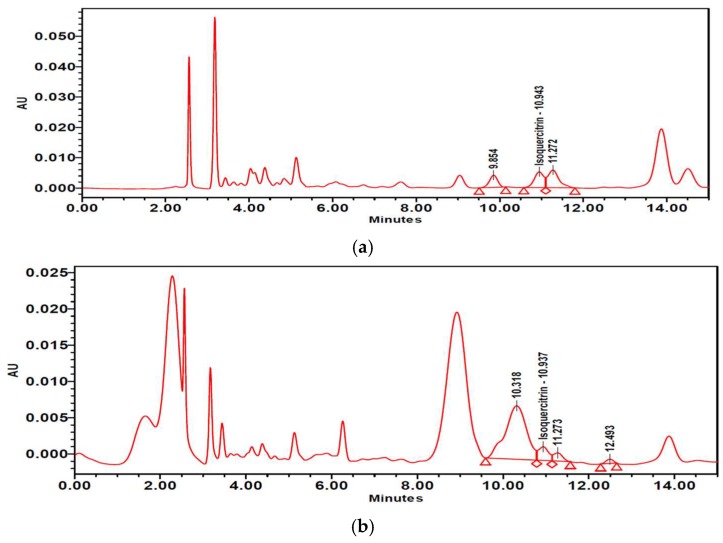

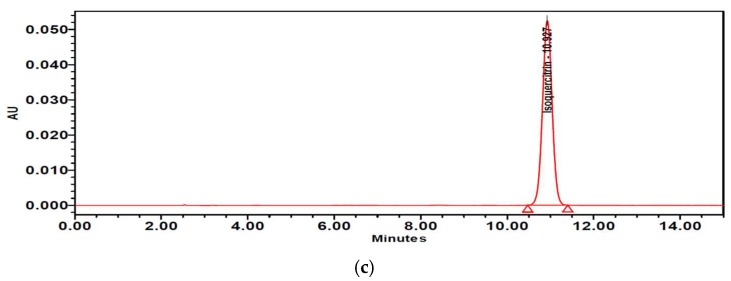
High-performance liquid chromatography (HPLC) chromatograms of (**a**) PTFE and (**b**) PTFFE. (**c**) Peak of the isoquercitrin standard.

**Figure 10:**
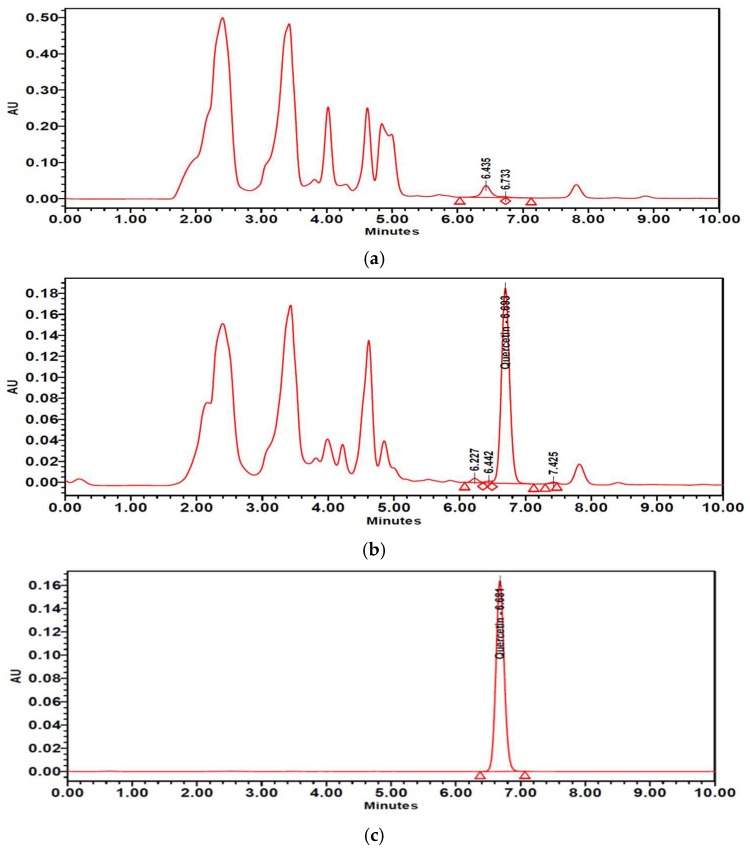
HPLC chromatograms of (**a**) PTFE and (**b**) PTFFE. (**c**) Peak of quercetin standard.

The contents of isoquercitrin and quercetin in PTFE and PTFFE were confirmed by HPLC analysis. The result showed that PTFE contained isoquercitrin (10.021 mg/g) and quercetin (1.442 mg/g). However, after fermentation, PTFFE, the isoquercitrin (3.715 mg/g) decreased and quercetin (114.134 mg/g) remarkably increased (Table 2, Figure 9 and Figure 10).

## 3. Discussion

B16 melanoma is a murine tumor cell line used for research as a model for the study of metastasis and solid tumor formation in human skin cancers. Although it is different from normal human melanocyte and human melanoma cell lines in various cellular behaviors [26,27,28], this cell line is also an excellent model for studying the effects of extracts/compounds on melanin production [29]. Numerous studies have found that upregulation of melanogenesis is often observed in malignant melanoma with overexpressed tyrosinase levels in blood as well as tumor tissues [30]. In addition, MITF has been reported as an amplified oncogene in human melanomas and displays functional roles in transcriptional gene expression related to survival, proliferation, cell cycle progression and chemoresistance [31]. Therefore, MITF or its target genes (e.g., tyrosinase) is clinically and cosmetically important for the treatment of melanoma and melanin-related skin diseases. In this study, we therefore evaluated the effect of PTFFE on melanogenesis in B16F10 cells. We also studied signaling pathways to understand the melanogenic mechanism in B16F10 cells. The results of our study indicated that PTFFE inhibited melanogenesis in B16F10 mouse melanoma cells. The effects of PTFFE on melanogenesis were evaluated by the melanin content and tyrosinase activity in the cells. In addition, western blot analysis was performed to determine the expression of TRP-1, TRP-2 and tyrosinase, as well as their regulator MITF. Furthermore, western blot analysis was used to confirm the phosphorylation of AKT and MAPKs, which are regulators of MITF phosphorylation. An MTT assay was performed to determine the range of PTFFE concentrations not causing cytotoxicity and the results indicated that concentrations of 62.5, 125 and 250 μM were safe for the cells (Figure 1).

Recent studies have reported that melanogenic enzymes (TRP-1, TRP-2 and tyrosinase) are regulated by MITF, which is a nuclear transcription factor [4,5,6]. Therefore, inhibition of MITF expression causes inhibition of the expression of melanin-related proteins, thereby reducing the melanin synthesis in B16F10 cells. The results of our study indicated that the expression of MITF as well as TRP-2 and tyrosinase was reduced in PTFFE-treated groups in a concentration-dependent manner compared with their levels in the group treated with α-MSH alone (Figure 4). These results suggested that PTFFE exerts antimelanogenic effects by decreasing the MITF expression.

It has been reported that the PI3K/AKT signaling pathway is related to melanogenesis in B16F10 cells [13]. Increased AKT phosphorylation has been shown to inhibit the MITF expression and downstream signaling pathways, thereby reducing activation of melanogenesis in α-MSH-induced B16F10 cells [32,33]. To investigate whether PTFFE modulates this signaling pathway, we evaluated the phosphorylation status of AKT by western blot analysis and also examined the tyrosinase activity in cells treated with LY294002 (an AKT inhibitor). The results showed that PTFFE increased the AKT phosphorylation, while tyrosinase activity induced in cells by α-MSH or LY294002 was reduced by PTFFE treatment (Figure 5 and Figure 6). These results indicated that PTFFE inhibited the tyrosinase activity through the AKT signaling pathway, thus effectively inhibiting activation of melanogenesis in α-MSH-induced B16F10 cells.

Previous studies have reported that MAPK signaling pathways are associated with melanogenesis. Thus, suppression of p38 and JNK phosphorylation decreased melanogenesis by downregulating MITF and the expression of melanogenic enzymes, while suppression of ERK phosphorylation enhanced the melanin production [10,11,12]. In this study, PTFFE increased the phosphorylation of ERK and decreased that of JNK and p38 (Figure 7). Thus, to examine whether the ERK signaling pathway is involved in melanogenesis, we evaluated the tyrosinase activity in the presence of PD98059 (an ERK inhibitor). The data indicated that the tyrosinase activity induced in B16F10 cells by α-MSH or both α-MSH and PD98059 was inhibited by PTFFE treatment (Figure 8). The results suggested that PTFFE exerted antimelanogenic effects in B16F10 cells via MAPK signaling pathways.

To determine the PTFFE constituents involved in antimelanogenesis, HPLC fingerprinting was performed using several standards and isoquercitrin and quercetin were identified. Quercetin is a well-known compound with antimelanogenic activity in B16F10 cells [34,35]. The chemical structure of isoquercitrin contains quercetin bound to a glycoside [36]. Fermentation is a metabolic process in which sugars are converted into acids, gases and alcohol [37], mainly with the participation of yeasts, bacteria and fungi. The result of HPLC showed that PTFE contained isoquercitrin (10.021 mg/g) and quercetin (1.442 mg/g). However, after fermentation, the isoquercitrin (3.715 mg/g) decreased and quercetin (114.134 mg/g) remarkably increased (Table 2; Figure 9 and Figure 10). These results may suggest that saccharification by microorganisms during fermentation may decompose glycosides of isoquercitrin and increase the amount of quercetin, which has a whitening effect.

The results of our investigation showed that PTFFE inhibited melanogenesis in B16F10 cells without cytotoxicity. In addition, the data indicated that PTFFE decreased p38 and JNK phosphorylation and increased that of ERK in MAPK signaling pathways. Furthermore, PTFFE reduced the MITF expression by enhancing AKT phosphorylation. It was also found that the amount of quercetin increased in the extract through PTFE fermentation. Moreover, the results of the human skin primary irritation test proved that PTFFE is safe on human skin without any severe side effects. Therefore, PTFFE may be considered a potential antimelanogenic candidate for topical applications. Further detailed studies will be necessary to confirm the inhibitory effects of PTFFE in melanogenesis using human participants.

## 4. Materials and Methods

### 4.1. Chemicals and Reagents

Dimethyl sulfoxide (DMSO), α-MSH, NaOH, MTT, radioimmunoprecipitation assay (RIPA) buffer and l-DOPA were obtained from Sigma–Aldrich (St. Louis, MO, USA). Dulbecco’s modified Eagle’s medium (DMEM), fetal bovine serum (FBS), penicillin/streptomycin, trypsin–ethylenediaminetetraacetic acid and PD98059 were purchased from Thermo Fisher Scientific (Waltham, MA, USA). Antibodies against tyrosinase, TRP-1, TRP-2 and MITF were purchased from Santa Cruz Biotechnology (Dallas, TX, USA). LY294002 and antibodies against p-p38, p38, p-JNK, JNK, p-ERK, ERK, p-AKT, AKT and β-actin were obtained from Cell Signaling Technology (Danvers, MA, USA). An enhanced chemiluminescence (ECL) kit and 2× Laemmli sample buffer were obtained from Biosesang (Sungnam, Gyeonggi-do, Korea) and Bio-Rad (Hercules, CA, USA), respectively.

### 4.2. P. tinctorium Flower Extract

*P. tinctorium* flowers were supplied by JejuIndi, Inc. (Jeju, Korea). Dried flowers (10 g) were mixed with 600 mL of a 70% ethanol solution and incubated at room temperature for 24 h. The extract was filtered through filter paper and the solvent was evaporated. After freeze-drying, the extract was powdered and used in experiments. The yield of the extract was 19.8% (1.98 g).

### 4.3. P. tinctorium Fermented Flower Extract

Equal amounts (0.26 g) of dried *P. tinctorium* flowers, red nuruk powder and sugar were added to 180 mL of distilled water and the mixture was fermented for 5 days in an incubator at 30 °C. Then, 420 mL of 95% ethanol was added and the mixture was extracted with 70% ethanol for 24 h. The solution was filtered and concentrated to prepare a powder. The yield of the extract was 44.87% (0.35 g).

### 4.4. High-performance liguid chromatography (HPLC) Analysis of P. tinctorium Flower Extract and Fermented Extract

Since quercetin was reported to be effective in antimelanogenesis [34,35], it was used as a standard compound for analysis of the PTFFE composition. The HPLC system used for the analysis was a 2695 separation module (Waters, Milford, MA, USA) and the HPLC conditions are shown in Table 3. Data analysis was performed using the Waters Empower software. Analytical samples, PTFE and PTFFE, were dissolved in 70% ethanol at a concentration of 10 mg/mL.

### 4.5. Cell Culture

B16F10 melanoma cells were purchased from the Korean Cell Line Bank (Seoul, Korea) and cultured in phenol red-free DMEM supplemented with 10% FBS and 1% penicillin/streptomycin in a humidified atmosphere containing 5% CO_2_ in air at 37 °C. Cells were treated with various concentrations of PTFFE and α-MSH (200 nM) and arbutin (100 μM) were used as controls.

### 4.6. Measurement of Cell Viability

Cell viability was measured by the MTT assay, as reported by Carmichael [38]. B16F10 cells were cultured in 24-well plates for 24 h and then treated with PTFFE at various concentrations (62.5, 125 and 250 μg/mL) for 48 h. Briefly, the MTT solution was added to the plate and then removed after incubation for 4 h. The formazan crystals formed were dissolved in 1 mL of DMSO. The absorbance was measured in a 96-well plate at 540 nm using an ELISA reader (Tecan, Mannedorf, Switzerland).

### 4.7. Lactate Dehydrogenase (LDH) Release Assay

B16F10 cells were seeded in 24-well plate and cultured 24 h and incubated with various concentrations of PTFFE (62.5, 125, 250, 500 and 1000 μg/mL) for 48 h. After incubation, 50 μL of the culture supernatant was transferred into a new 96-well plate. LDH released to the extracellular medium was quantified by the CytoTox 96 Non-Radio Cytotoxicity Assay (Promega, Madison, WI, USA) according to the manufacturer’s instructions. Fresh culture medium was used as blank. The absorbance was measured at 490 nm using an ELISA reader.

### 4.8. Measurement of Intracellular Melanin Content

Cells were treated in a 6-well plate with PTFFE (62.5, 125 and 250 μg/mL), α-MSH (200 nM) and arbutin (100 μM) for 72 h at 37 °C. After DMEM was removed, the cells were washed twice with phosphate-buffered saline (PBS) and then treated with 1 N NaOH for 1 h at 80 °C to dissolve cell pellets. The experiment was carried out at least three times and melanin contents were measured at 405 nm using an ELISA reader.

### 4.9. Measurement of Intracellular Tyrosinase Activity

B16F10 cells (1.5 × 10^5^ cells/dish) were seeded in 100-mm dishes and cultured for 24 h. Subsequently, the cells were treated with PTFFE (62.5, 125 and 250 μg/mL), α-MSH (200 nM) and arbutin (100 μM) for 72 h at 37 °C, 5% CO_2_ under humidified conditions. The supernatants were removed and the cell pellets, collected into e-tubes, were lysed with RIPA buffer containing a 1% protease inhibitor cocktail. After centrifugation for 30 min at 15,000 rpm, the supernatant was collected into another e-tube and the protein level was quantified using a bicinchoninic acid assay (BCA) kit. The protein concentrations in the lysates were adjusted to the same values using RIPA buffer. Next, 20 μL of an adjusted protein sample and 80 μL of l-DOPA (2 mg/mL) were added to a 96-well plate to measure tyrosinase activity. After incubation at 37 °C for 2 h, absorbance was measured at 490 nm using an ELISA reader.

### 4.10. Western Blot Analysis

B16F10 cells were incubated with α-MSH (200 nM), arbutin (100 μM) and various concentrations of PTFFE (62.5, 125 and 250 μg/mL). Afterward, the cells were washed twice with PBS and collected into 1.5-mL e-tubes, followed by lysis in RIPA buffer containing a 1% protease inhibitor cocktail for 1 h. After centrifugation at 15,000 rpm for 15 min at 4 °C, the supernatant was collected into another e-tube. The protein concentrations were measured in the cell lysates using the BCA kit. After heating the samples at 100 °C for 5 min, equal amounts of protein (in 20 μL) were loaded on 10% sodium dodecyl sulfate polyacrylamide gels, which were run for 1 h at 150 V. Then, the proteins were transferred to a polyvinylidene difluoride membrane. The membrane was washed six times with Tris-buffered saline (20 mM Tris base, 137 mM NaCl, pH 7.6) containing 0.1% Tween 20 (TBST), then blocked with TBST containing 5% skim milk for 1 h and incubated overnight at 4 °C with primary antibodies diluted in TBST (1:1000). Afterward, the membranes were washed several times with TBST and incubated with a secondary antibody, rabbit anti-mouse IgG (1:3000), for 1 h, followed by washing several times with TBST. Target proteins were detected using an ECL kit.

### 4.11. Human Skin Primary Irritation Test

A human skin irritation test with PTFFE was performed by the clinical trial center for bio-industry at Semyung University. The subjects participating in the test had an average age of 40.2 years, with 13% in their 20 s, 20% in their 30 s, 57% in their 40 s and 10% in their 50 s. The total number of the subjects was 30. The subjects were kept clean and dry to ensure the same measurement conditions. The skin was stabilized at the temperature of 22 ± 2 °C and humidity of 40–60% for at least 30 min. The test area was wiped with 70% ethanol and then dried. After that, 25 μL of PTFFE (250 μg/mL) was applied to the test area of the subject and fixed with a patch, which was removed after 48 h. After removing the patch, the test area was marked with a marking pen and the test site was observed after 30 min and 24 h. Skin responses were evaluated according to the criteria of the International Contact Dermatitis Research Group. All assessments were performed under standard lighting conditions by a qualified research expert or dermatologist. This study was approved by the ethics committee of the clinical trial center for bio-industry at Semyung University (Chungbuk, Korea).

### 4.12. Data Analysis

All experimental results are expressed as the mean ± SD of at least three independent experiments. The results were analyzed using a Student’s *t*-test. Statistical significance was considered at *p* < 0.01 (**) or *p* < 0.001 (***).

## Figures and Tables

**Table 1:** Results of the human skin primary irritation test.

No.	Test Material	48 h					72 h					Reaction Grade ^1^		
		±	1+	2+	3+	4+	±	1+	2+	3+	4+	48 h	72 h	Mean
1	Control	− ^2^	−	−	−	−	−	−	−	−	−	0	0	0
2	PTFFE (125 μg/mL)	−	−	−	−	−	−	−	−	−	−	0	0	0
3	PTFFE (250 μg/mL)	−	−	−	−	−	−	−	−	−	−	0	0	0
4	PTFFE (500 μg/mL)	−	−	−	−	−	−	−	−	−	−	0	0	0

^1^ Reaction grade = ∑[{Grade × No. of Responders}/{4(Maximum grade) × 30(Total Subjects)}] × 100 × (1/2). ^2^ No reaction.

**Table 2:** Contents of isoquercitrin and quercetin in PTFE and PTFFE.

Compound	PTFE (mg/g)	PTFFE (mg/g)
Isoquercitrin	10.021	3.715
Quercetin	1.442	114.134

**Table 3:** HPLC conditions.

Standard	Run Time (min)	Injection Volume (µL)	Wavelength (nm)	Column Temperature (°C)	Flow Rate (mL/min)	Solvent Ratio (A:B)
Isoquercitrin	15	5	355	30	1.2	2:8
Quercetin	10	10	280	37	1.0	4:6
Parameter			Condition			
Column			YMC-Triart C18 (250 × 4.6 mm, 5 μm, 12 nm)			
Mobile phase			A: Acetonitrile, B: 20 mM Phosphoric acid in distilled water			
Sample Temperature			15 °C			
Sample Concentration			10 mg/mL			
Detector			Photodiode array (Waters)			
Separation			Waters 2695			
